# *From Bench to Bedside*: Evaluation of AHCC Supplementation to Modulate the Host Immunity to Clear High-Risk Human Papillomavirus Infections

**DOI:** 10.3389/fonc.2019.00173

**Published:** 2019-03-20

**Authors:** Judith A. Smith, Lata Mathew, Anjali Gaikwad, Barbara Rech, Maryam N. Burney, Jonathan P. Faro, Joseph A. Lucci, Yu Bai, Randall J. Olsen, Teresa T. Byrd

**Affiliations:** ^1^Department of Obstetrics, Gynecology and Reproductive Sciences, UTHealth McGovern Medical School, Houston, TX, United States; ^2^Department of Pharmacy, Memorial Hermann Cancer Center, Houston, TX, United States; ^3^UT Physicians Women's Center, Houston, TX, United States; ^4^Specialists in Obstetrics & Gynecology, Houston, TX, United States; ^5^Department of Pathology, UTHealth McGovern Medical School, Houston, TX, United States; ^6^Department of Molecular Pathology, Institute for Academic Medicine, Houston Methodist Research Institute, Houston, TX, United States

**Keywords:** AHCC, HPV, prevention, nutritional supplementation, cervical cancer

## Abstract

**Objective:** There is currently no effective medicine or supplement for clearance of high risk- human papillomavirus (HR-HPV) infections. We have taken a systematic approach evaluating the potential use of AHCC supplementation to support clearance of HR-HPV infections. The primary objective of this research was to evaluate AHCC supplementation to modulation of the host immune system to clear HR-HPV infections from bench to bedside.

**Methods:** Cervical cancer cells, CaSki (HPV16^+^), HeLa(HPV18^+^), SiHa(HPV16/18^+^), and C-33A(HPV^−^), were treated *in vitro* with AHCC 0.42 mg/mL daily x7 days then observed x7 days with daily sample collection. A confirmatory study in cervical cancer mouse models, SiHa(HPV16/18^+^) and C-33A(HPV^−^), was conducted: mice were divided into three groups per cell line then dosed with AHCC 50 mg/kg/d (*N* = 10), or vehicle alone (*N* = 10), or no supplementation (*N* = 10) for a total of 90 days followed by 30 days of observation. Tumors were measured 3x/week and blood samples collected bi-weekly to evaluate interferon (IFN) alpha(α), beta(β), and gamma(γ) and immunoglobulin G(IgG) by immunoassays. Tumors were evaluated for HR-HPV expression by PCR. Two pilot studies of 10 patients each were conducted in women with confirmed persistent HR-HPV+ infections. The 1^st^ study evaluated AHCC 3g from 5 weeks up to 6 months and 2nd study evaluated AHCC 1g < 8 months. HR-HPV DNA status and the immune panel were monitored at each visit.

**Results:** HR**-**HPV clearance was observed *in vitro and* confirmed in the animal studies as a durable response. Four of six (66.7%) patients had confirmed HR-HPV clearance after 3–6 months of AHCC 3g. Similarly, 4 of 9 (44%) patients had confirmed HR-HPV clearance after 7 months of AHCC 1g. Suppression of IFNβ <25 pg/mL was observed in those clearing the HR-HPV infection.

**Conclusion:** Pre-clinical *in vitro* and *in vivo* studies demonstrated durable clearance of HR-HPV infections. The preliminary data from the two pilot studies suggested that AHCC supplementation supports the host immune system for successful clearance of HR-HPV infections. A confirmatory phase II randomized, double-blinded, placebo-controlled study is ongoing.

## Introduction

Worldwide, cervical cancer is the fourth most common malignancy in women and a major cause of morbidity and mortality (1). It accounts for nearly 10% of all cancers, and ~265,700 women die from this disease every year worldwide (1). The etiology of cervical cancer has been identified and confirmed associated with high risk-human papillomavirus (HR-HPV) (2–5). The human papillomavirus (HPV) is classified as a non-enveloped, double stranded DNA virus that generally infects the epithelial layer of cells including cutaneous and mucosal surfaces and associated with benign warts, carcinoma *in situ* and ultimately malignant lesions (6, 7). When HR-HPV infections persist overtime, patients have an increased risk of developing cervical cancer (8).

The proprietary, standardized extract of cultured Lentinula edodes mycelia, AHCC (Amino Up Chemical Co. Ltd, Sapporo, Japan), was developed in Japan in 1992. Several studies have reported a variety of therapeutic effects, including antioxidant and anticancer activity and improvement of immune response (9, 10). In animal studies, AHCC has shown the ability to treat and prevent cancer, and modulate the immune system to prevent infectious processes (9–11). Gao et al. demonstrated the immunomodulating effects of AHCC in a study that showed enhanced antigen (Ag) activation of CD4 (+) and CD8 (+) T cells, and the increased frequency of tumor Ag-specific IFN-gamma producing CD8 (+) T cells as well as increases in cell numbers of NK cells and gamma delta T cells (10). In clinical studies AHCC has demonstrated benefit to decrease risk of infection and ameliorate symptoms of existing infections (9–11, 13). Since studies have demonstrated AHCC induces apoptosis (12–14), it is possible that AHCC may also prevent/delay tumor growth regardless of the role of HR-HPV.

This manuscript presents over a decade of research from bench to bedside to test the hypothesis that AHCC supplementation will modulate the host immune system to effectively clear chronic, persistent HR-HPV infections. There is currently no cure for persistent HR-HPV infections. Hence, confirmation of preclinical findings was essential before reporting of this data to demonstrate translation to humans. First preclinical study objectives were to evaluate if AHCC supplementation would clear HR-HPV expression *in vitro* and *in vivo* cervical cancer mouse models, and to define the mechanism of clearance of HR-HPV infections with AHCC supplementation. In the initial pilot studies, objectives were to evaluate if AHCC supplementation would modulate the host immune system to clear HR-HPV infections in women with confirmed, persistent HR-HPV infections, to determine duration of AHCC supplementation needed to clear HPV infections and confirm durability of response.

## Materials and Methods

### Preclinical Studies

#### Chemicals and Reagents

AHCC was generously provided by Amino Up Chemical Co, Ltd. (Sapporo, Japan). Fetal bovine serum (FBS) and trypsin-EDTA were purchased from GIBCO Invitrogen Co. (Carlsband, CA). The 3-(4,5-dimethylthiazole)-2,5-diphenyl tetrazolium bromide (MTT) and dimethyl sulphoxide (DMSO) were purchased from Sigma-Aldrich Co. (St. Louis, MO). Ready–to–use sandwich ELISA kits for the detection of mouse IFN-alpha/beta, IFN-gamma were purchased from eBioscience (San Diego, CA). ELISA kit for the detection of mouse IgG1 was purchased from GenWay Biotech, Inc. (San Diego, CA).

#### Cell Culture

All human cervical cancer cell lines C-33A (HPV negative) and CaSKi (HPV16 positive), HeLa (HPV 18 positive), and SiHa (HPV16 & HPV18 positive) were obtained from the American Type Culture Collection (ATCC, Manassas, VA). The SiHa squamous cell carcinoma, HeLa adenocarcinoma, and C-33A carcinoma cell lines were propagated in a media consisting of EMEM with 2 mM L-glutamine and Earl's BSS adjusted to contain 1.5 g/L sodium bicarbonate, 0.1 mM non-essential amino acids, 1.0 mM sodium pyruvate and 10% FBS. The CaSKi epidermoid carcinoma cell line was propagated in a media consisting of RPMI 1640 with 2 mM L-glutamine adjusted to contain 1.5 g/L sodium carbonate, 4.5 g/L glucose, 10 mM HEPES, 1.0 mM sodium pyruvate and 10% FBS. All cell lines were grown in 75-cm^2^ culture flasks in 5% CO_2_ in air at 37°C to 90% confluence. Cell lines used for this study were maintained for <15 passages to prevent major changes in cell line characteristics.

#### Drug Solutions

AHCC 40 mg/mL stock solution was prepared by dissolving 600 mg of AHCC powder in 15 mL phosphate buffered saline (PBS) and filters sterilized with 0.2 μm syringe filter. All additional dilutions were completed as required with the respective cell culture media for each cell line. The MTT stock solution was prepared by dissolving 54 mg of MTT in 20 mL PBS to achieve a final concentration of 0.3 mg/mL. Fresh standards and dilutions were made for each experiment.

#### *In vitro* AHCC Study to Evaluate Clearance of HR-HPV:

Growth inhibition assays were conducted as previously described (12). The IC_20_ (inhibitory concentration to achieve 20% cell death), and IC_50_ for AHCC and each cell line were calculated (Table 1). During the designing and planning of this pre-clinical research study, the achievable AHCC human systemic plasma concentration data was unavailable to investigators. Based on the current recommended dosage of 3 g total daily dose as instructed by the manufacturer, assuming 100% bioavailability and using an estimated total blood volume of an average adult of seven liters the highest achievable plasma concentration would be 0.42 mg/mL which was below the IC_20_ concentration so would not by cytotoxic to the cell lines. Hence for all of the *in vitro* studies, the 0.42 mg/mL concentration was selected as an estimate of the clinically relevant concentration. All experiments were done in quadruplicate. Five million cells of SiHa (HPV 16/18 positive) and C-33A (HPV negative) were treated in T25 flasks, one time with AHCC (0.42 mg/mL) for 72 h (Table 1). The IC_20_ concentration was selected to maintain cell viability, yet high enough concentration of AHCC to “clear” the HR-HPV infection. Untreated cells served as a control. Cells were harvested at 24, 48, and 72 h time periods. The same experiment was repeated for 7 days with addition of fresh AHCC (0.42 mg/mL) every 24 h, then observed for an additional 7 days. Untreated cells served as a control. Cells were harvested once every 24 h for total of 14 days during supplementation and observation phases of the study. DNA was extracted from all samples and used for PCR as described below.

**Table 1:** Summary of *in-vitro* cytotoxicity: Four cervical cancer cell lines were supplemented with AHCC.

**AHCC (mg/mL)**	**C-33A**	**SiHa**	**HeLa**	**CaSKi**
IC_20_	0.48	0.82	0.77	0.75
IC_50_	3.3	2.3	4.0	3.8

#### Human Cervical Cancer Mouse Models

There currently is not an ideal mouse model for evaluating HR-HPV infections alone. A cervical cancer model was selected as best surrogate for evaluating elimination of HR-HPV infections because the SiHa human cervical cancer cell line expresses both HPV16 and HPV18. The C33 tumor model was selected as a HPV negative control for the study. The protocol was reviewed and approved by the institutional animal welfare committee (AWC) prior to initiating any animal work. For this study, 70 female athymic/immune-suppressed mice, 6–8 weeks old, were obtained from Harlan Laboratories (Houston, Texas). All the mice weighed about 22–26 g; they were maintained five per cage in specific pathogen free (SPF) barrier room, with a temperature of 22 ± 3 and 45 ± 3°C RH%. There was free access to autoclaved food and reverse osmosis autoclaved water. The experiment procedures and the handling of the mice were in strict accordance with the guide for the care and use of laboratory animals. There was a group of healthy controls which had 10 athymic/immune-suppressed mice in it, they were not treated throughout the study nor were they injected with any cancer cell lines and served as controls for immune marker monitoring. The remaining mice were divided into three groups of 10 for each cell line (*N* = 60 for two cell lines). There were three arms of this study: AHCC supplementation arm (*N* = 10), no supplementation control arm (*N* = 10) and a vehicle (autoclaved water) control arm (*N* = 10). The no supplementation control arm had no study intervention; Following traditional cancer chemoprevention models, the AHCC supplementation arm received an oral dose (50 mg/kg, in 0.25 mL, gastric gavage) and the vehicle control arm received an oral dose of autoclaved water (0.25 mL, gastric gavage) per day starting on day zero and continued until completion of the study (day 90) to evaluate if AHCC also has role in prevention of cancer growth. The dose of AHCC was selected based current estimated maximum recommended dosage of 3 g total daily dose as instructed by the manufacturer, assuming 100% bioavailability and using an estimated total blood volume of an average adult of 60 kg. It is also the dose used in previous AHCC studies in combination with chemotherapy. SiHa cells (0.5 × 10^6^) and C33A cells (0.5 × 10^6^) were dispersed in PBS with 20% matrigel and injected subcutaneously in female athymic/immune-suppressed mice on day 8 after 1 week of AHCC supplementation. Each mouse grew one tumor on the dorsal surface. Tumor measurements were obtained three times per week with electronic Vernier calipers (Mitutoyo, Utsunomiya, Japan). Mice were monitored daily for signs/symptoms of morbidity including but not limited to lethargy, weight loss, anorexia, or hunching. Mice were sacrificed via CO_2_ inhalation followed by cervical dislocation, when tumor diameter was >15 mm^2^, and if a 10% or greater decrease in body weight was found during the study period. Each group was subdivided into two groups A & B. Blood sample of 1% of the body weight, from all the mice of all groups were collected from their facial vein every 14 days in an EDTA containing tube, to carry on the assay for expression of different immune markers at the end of the study. The whole blood was centrifuged; the separated serums were collected in a tube and were stored at negative 20°C freezer. The serums of sub group A were tested for IgG1 and IFN γ, and the serums of sub group B were tested for IFNα/β. At the end of the study all the remaining mice were sacrificed, blood samples were collected by cardiac puncture, serums were separated, and stored at negative 20°C. Tumors were collected in cryovials and were stored at negative 80°C for further study in PCR studies to evaluate HR-HPV status as described below.

#### PCR-Based Analysis of HPV (16 or 18) DNA:

DNA extraction was done from treated and untreated cell samples as well as mouse tumor samples using Promega DNA extraction Kits (Madison, WI) according to manufacturer's protocol. The polymerase chain reaction (PCR) was used to detect the expression of human papilloma virus (HPV) gene as per the manufacturer's protocol using Promega-Master Mix Kit (Madison, WI). The analyses were performed on 500 ng of purified DNA of treated and untreated cell and tumor samples as described below. PCR reaction contained 500 ng of DNA, 4 μL of Master Mix, and 4 μL of each consensus primer (MY11, MY09, β-actin reverse and β-actin forward). Thirty cycles of start denaturation (95°C for 4 min), denaturation (94°C-1 min), annealing (55°C-1 min), and extension (72°C-1.5 min) were performed on a BioRad thermocycler. β-actin was used as the DNA loading control. After the completion of the reaction, 10 μL each of all PCR products were resolved on 1.5% agarose gel with ethidium bromide against 100 bp DNA ladder. The image was captured under UV illumination using Kodak Imaging Station (Kodak, Rochester, NY). The primer sequence of MY11 is DNA 5′ to 3′-GCM CAG GGW CAT AAY AAT GG and MY09 is DNA 5′ to 3′-CGT CCM ARR GGA WAG TGA TC. The sequence of β-actin forward primer is AAC TGG GAC GAC ATC GAG AA and the sequence of β-actin reverse primer is AGA GGC GTA CAG GGA TAG CA.

#### ELISA for the Detection of Immune Markers

Following the respective time point supplementation regimens of base line, week 2, week 4, week 6 and end time point, blood samples were collected from mice and serum was separated and stored at −20°C for ELISA assays for the detection of IgG, IFNα/β, and IFNγ. For each series of immune markers determinations, a standard curve was constructed with known concentrations of these markers according to the manufacturer's protocol. Sandwich ELISAs for the detection of total IgG, IFN α/β, and IFN γ were performed according to the manufacturer's protocol. Serum concentrations of these three markers were calculated from standard graphs and were compared with base line concentrations. The percent increase and/or decrease in the concentration of IgG, IFNα/β, and IFNγ were calculated.

### Clinical-Studies

The pilot studies and study amendments (HSC-MS-12-0851) were approved by the Institutional Review Board of the University of Texas Health Science Center at Houston. Two pilot studies were conducted in women over the age of 30 with documented persistent HR-HPV infections for over 2 years or more, had laboratory values all within normal limits, normal histology up to CIN2 as documented by the primary gynecologist's records, and deemed otherwise healthy were enrolled on study. All patients signed informed consent to participate in each of the respective pilot studies. Patient demographic information was collected including: number of lifetime sexual partners, contraception methods, and periodic pregnancy testing throughout the study. Participants received either AHCC 1g by mouth once daily on an empty stomach for 6 months up to 8 months or AHCC 3g by mouth once daily on an empty stomach for 5 weeks up to 6 months to clear HR-HPV. HR-HPV testing was completed with Cervista HPV DNA testing (APTIMA, Hologic, Bedford, MA) in the AHCC 3g study. In the AHCC 1g study the patient samples were initially tested with HPV E6/E7 RNA testing (APTIMA, Hologic, Bedford, MA) and then all HPV RNA negative results confirmed or COBAS HPV DNA testing (Roche Molecular Systems, INC, Branchburg, NJ). A durable response was defined on remained HR-HPV negative by both HPV DNA and HPV RNA methods for at least 3 months during the observation period after stopping the AHCC supplementation. In both studies immune markers including IgG and IFN α/β/γ were monitored with ELISA assays as described above.

#### Data and Statistical Analysis

##### pre-clinical-studies

The “resource equation” method was employed to determine animal study sample size estimation (https://www.ncbi.nlm.nih.gov/pmc/articles/PMC3826013/). Briefly, an *E*-value that is equal to number of animals minus number of groups is calculated and ensured to be between 10 and 20. In this study there was independent comparisons of HR-HPV status as well as immune response between group 1(healthy control) vs. 2 (AHCC), 1 vs. 3 (vehicle control) and 1 vs. 4 (no supplementation control), thus *E* = (10 × 2)−2 = 18, which is within range. The student *t*-test analysis was used to evaluate the differences between the study arms that responded verses those that did not with *p*-values less than or equal to 0.05 considered significant.

##### clinical-study

The primary outcome for this study was the supplementation success rate, defined as the proportion of women free of HR-HPV infection at 6 months following initiation of supplementation. In this patient population, women with persistent HR-HPV infections, the expected clearance of HR-HPV infection on its own is zero to 10% (15). Persistent infection was defined by being HR-HPV positive consistently for >2 years that was confirmed by three or more consecutive HR-HPV positive tests results at least 6 months apart for more than 2 years. Since there is currently no active supplementation to clear HR-HPV infections, clinically any improvement in the response/clearance of HR-HPV infection in these women with persistent infections would be clinically significant. For this pilot study to determine if feasible and worth additional formal study, our target supplementation success rate was 25% or more. At a 0.05 confidence level, sample size was determined and its associated statistical power. The expected accrual was a minimum of 10 patients and a maximum of 25 patients to this study, and then follow patients for 1 month (28 days) after completion of supplementation. Patients who tested positive after 6 months of supplementation were considered supplementation failure. The student *t*-test analysis was used to evaluate the differences between the patients that responded verses those that did not with *p*-values less than or equal to 0.05 considered significant.

## Results

In the growth inhibition assays supplementation with AHCC for 72 h did not achieve any cell growth inhibition in any of the four human cervical cancer cell lines tested at clinically relevant concentration of 0.42 mg/mL. However, potential direct cytotoxicity was observed the concentration to achieve 50% growth inhibition (IC_50_), for AHCC ranged from 2.3 to 4 mg/mL in the four human cervical cancer cell lines however this is well above clinically achievable systemic concentrations. These results are summarized in Table 1.

**Figure 1:**
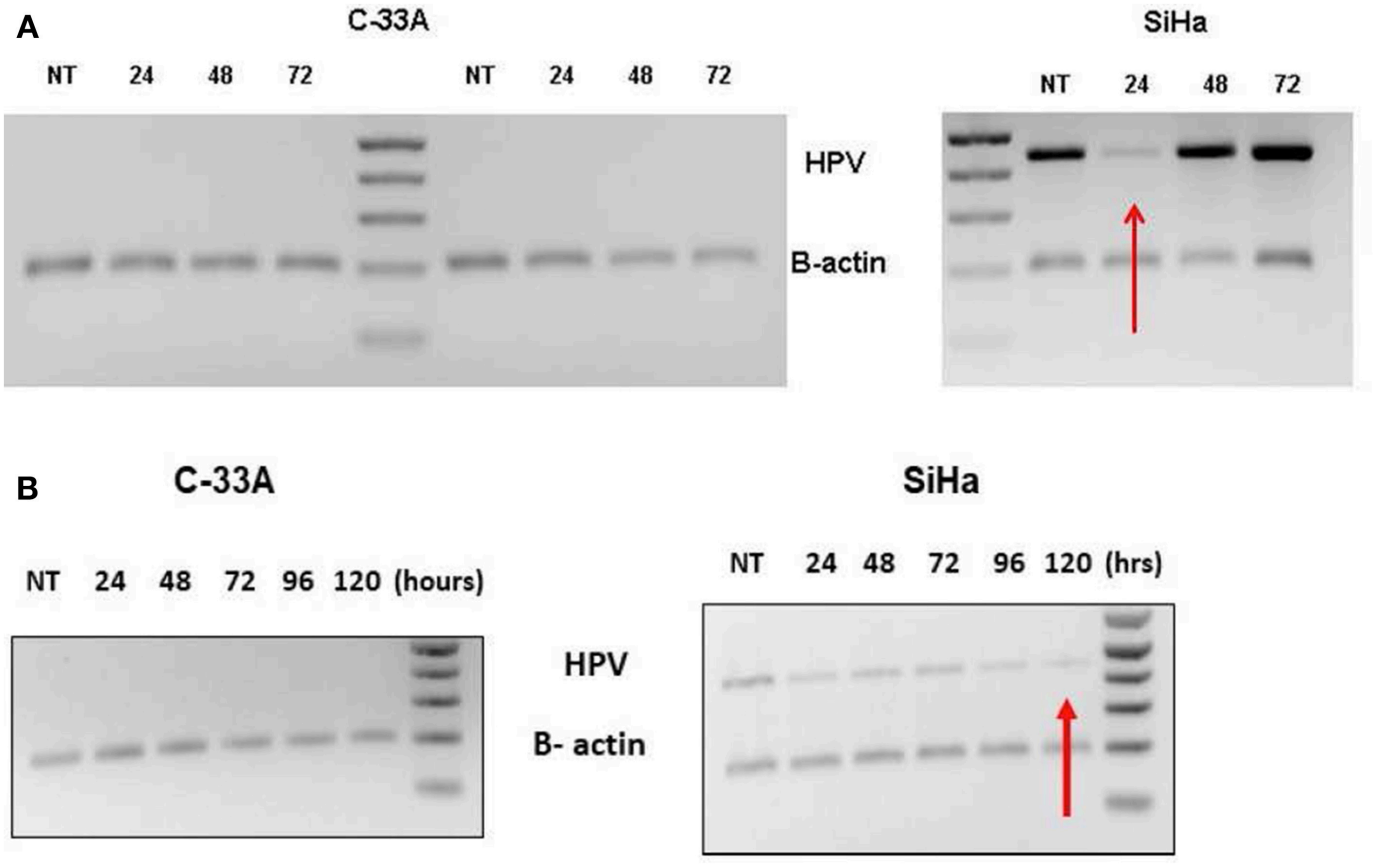
**(A)** The C-33A (HPV-) and SiHa(HPV 16/18+) human cervical cancer cell lines were supplemented *in vitro* with a one-time dose of AHCC 0.42 mg/mL and then incubated for 72 h. The study was completed in duplicate as described in materials and methods. While HR-HPV was suppressed for 24 h, it was not cleared evident by expression at 48 and 72 h. NT, non-treatment control. **(B)** The C-33A (HPV-) and SiHa(HPV16/18+) human cervical cancer cell lines were supplemented *in vitro* with daily fresh dose of AHCC 0.42 mg/mL once every 24 h for 5 days (120 h) as described in materials and methods. NT, non-treatment control. This was successful to eliminate HR-HPV expression in the SiHa cell line.

Supplementation with AHCC 0.42 mg/mL one-time dose for 72-h incubation, suppressed HR-HPV expression with first 24 h, but then HR-HPV expression was recovered by 48 h. The results for the C33a (negative control) and SiHa (HPV16 & HPV18 positive) cell lines are summarized in Figure 1A. Supplementation with AHCC 0.42 mg/mL once every 24 h for seven consecutive days followed by 7 days of observation with no supplementation cleared HR-HPV expression in all four of the HR-HPV+ cervical cell lines. The representative results in the SiHa (HPV16^+^/18^+^) compared to C33a HPV negative control are summarized in Figure 1B.

**Figure 2:**
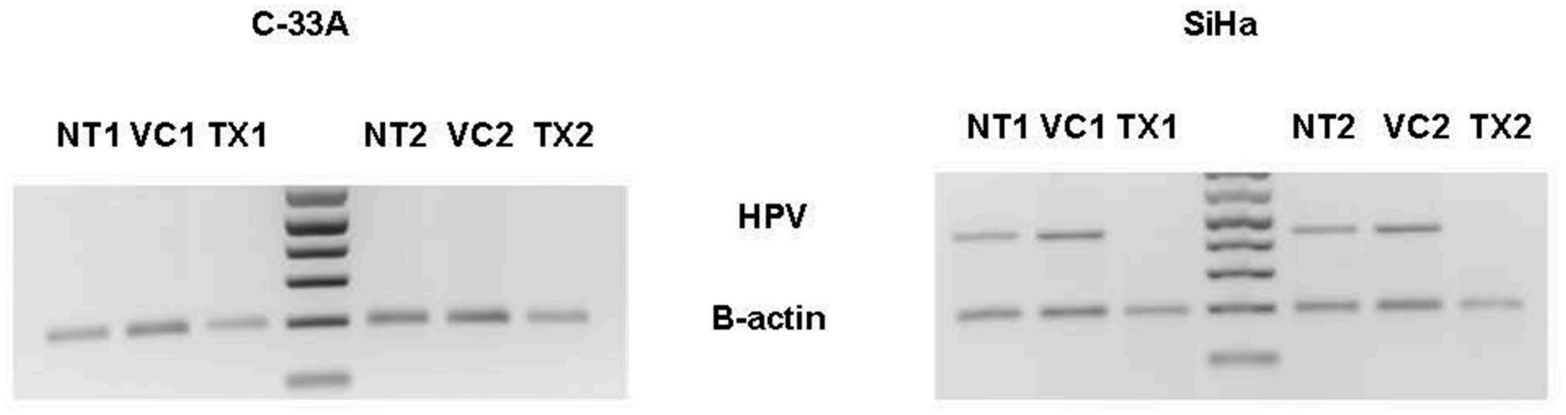
There were three arms in the animal study: AHCC supplementation arm (*N* = 10), no supplementation control arm (*N* = 10) and a vehicle (autoclaved water) control arm (*N* = 10). The AHCC supplementation arm received an oral dose (50 mg/kg, in 0.25 mL, gastric gavage); the no supplementation control had no study intervention, and the vehicle control arm received an oral dose of autoclaved water (0.25 mL, gastric gavage) per day starting on day zero and continued until completion of the study (day 90). TX1 represents at end of treatment and TX2 represents study day 90 which was 30 days after stopping AHCC supplementation. The absence of HR-HPV in the SiHa cell lines at TX1 and TX2 confirmed clearance of HR-HPV. NT, non-treatment control; VC, vehicle/placebo control.

**Figure 3:**
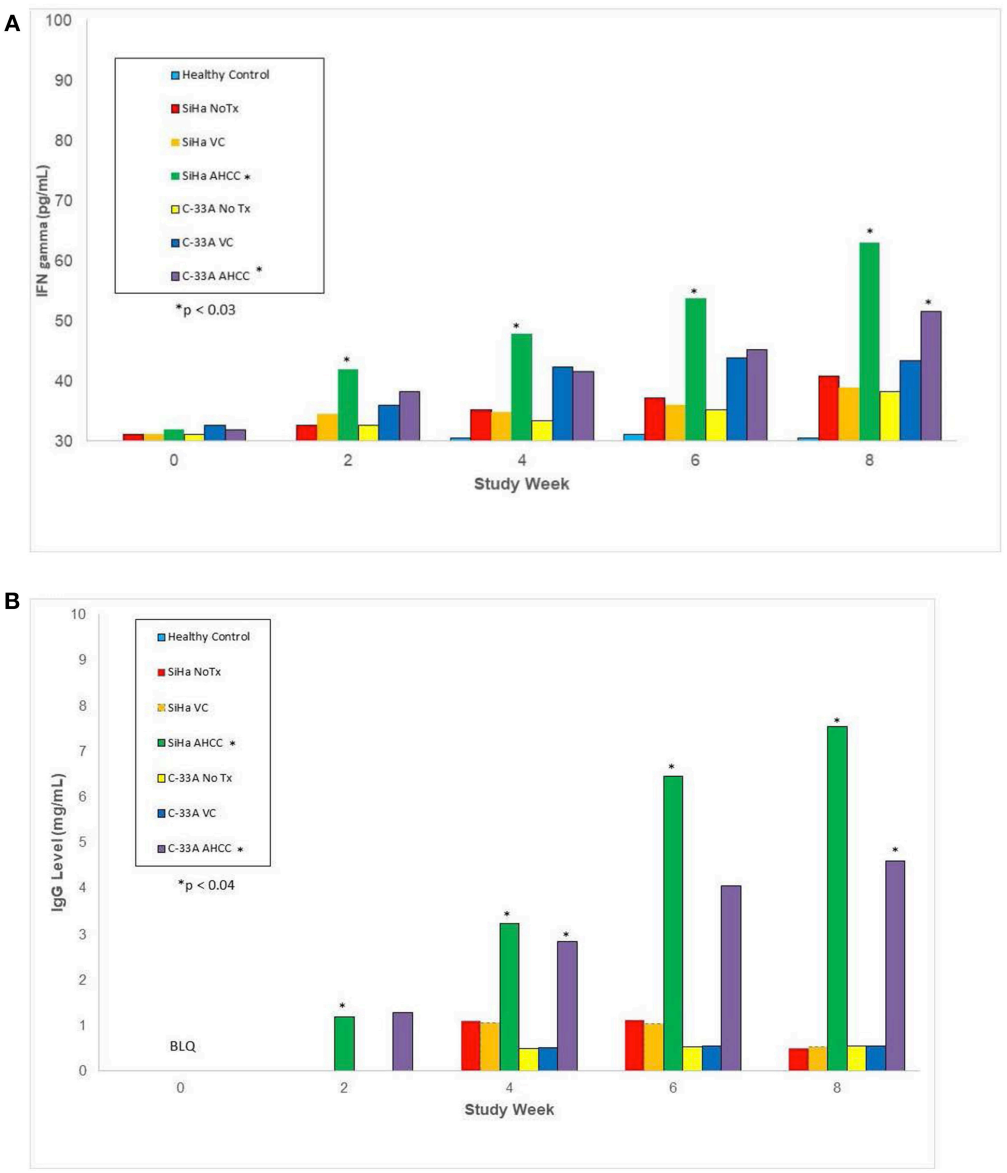
This represents immune marker data from animal studies including the IFN-γ, type II interferon associated with clearing viral infections, and IgG, antibody associated with immune response to viral infections, levels after 30 days AHCC 50 mg/kg supplementation once daily in mouse models as described in methods and materials. AHCC daily supplementation on IFN-γ and IgG levels increased with time and correlated with successful clearance of HR-HPV infections in the SiHa (HPV 16/18+) mouse model. **(A)** IFN-γ level response (NOTE: y-axis starts at 30 pg/mL) *p* < 0.03 **(B)** IgG1 level response, *p* < 0.04.

**Figure 4:**
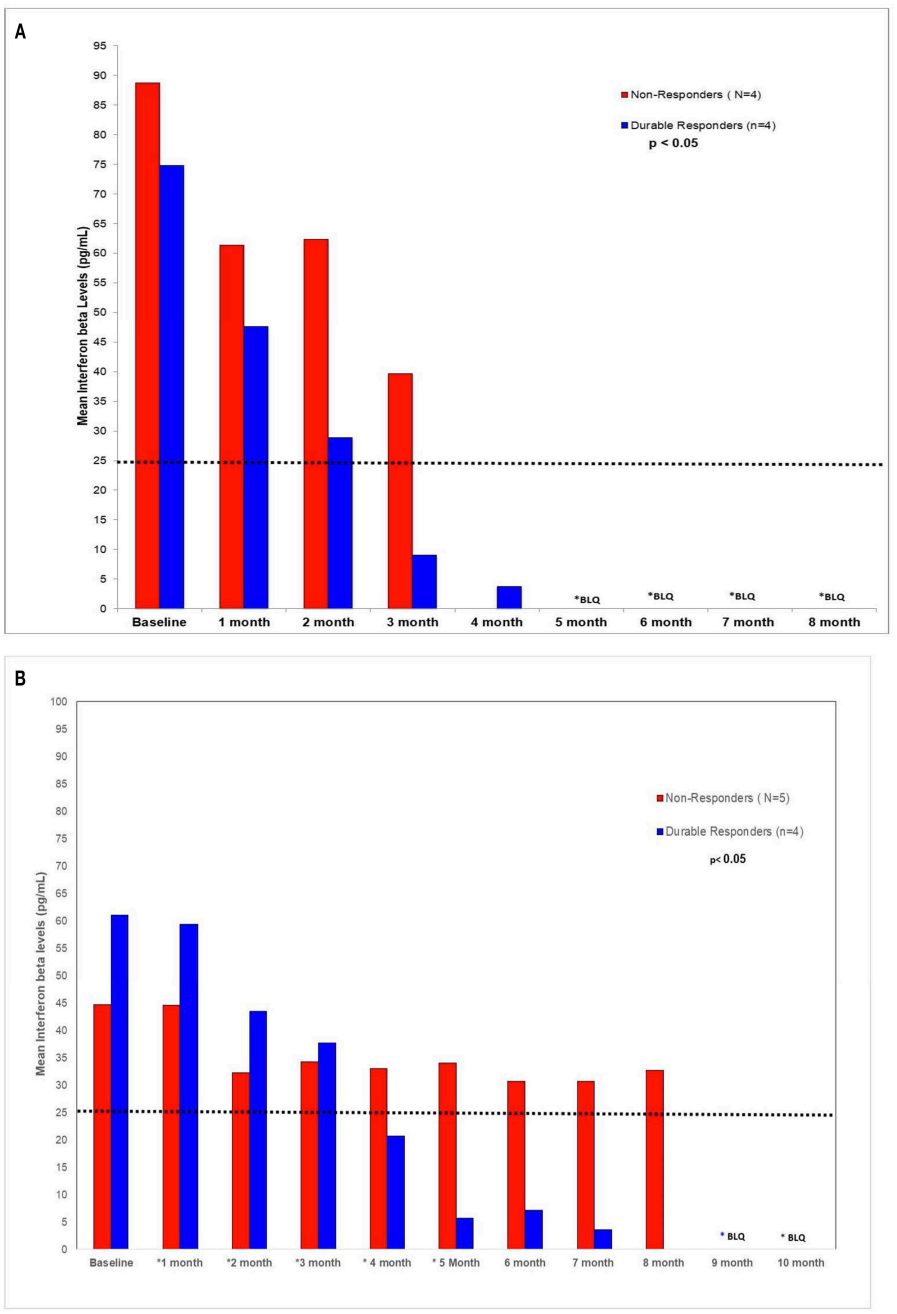
**(A)** AHCC 3g supplementation **(B)** AHCC 1g supplementation. IFN-β is a Type-1 IFN often associated with virulence of chronic viral infections. Chronic viral infections are often associated with high levels of IFN-β that leads to suppression of the production of IFN-γ and NK/T-cell cytotoxic cell immunity. It was observed in both pilot studies AHCC supplementation suppression of host IFN-β levels below level of 25 pg/mL that lead to modulation of the cytotoxic cell immunity to a successful, durable clearance of persistent, high risk HR-HPV infections.

In the *in vivo* confirmatory mouse studies, AHCC 50 mg/kg by mouth once daily for 90 days was associated with clearance of the expression of HR-HPV that was sustained after 30 days off supplementation. These results are summarized in Figure 2. Since the tumors were infected with HR-HPV and not the mouse, interferon beta levels that are typically elevated in chronic infections were normal. Impact of AHCC daily supplementation in the mice studies focused on IFN γ and IgG levels that represented the Type II interferon and immune response increased with time and correlated with successful clearance of HR-HPV infections in the human cervical cancer mouse model. These results are summarized in Figures 3A,B. In the first AHCC 3g pilot study, initially patients enrolled on study received AHCC 3g once daily on an empty stomach for up to 5 weeks returning once a week for HR-HPV testing and research blood sampling for immune markers. Once they tested negative, the AHCC was stopped and HR-HPV testing repeated after off the AHCC supplementation. Based on data review the supplementation plan was modified to continue AHCC 3g once daily on an empty stomach with once monthly HR-HPV testing and blood sampling with a required minimum of 1 month of AHCC supplementation after the first negative result. Based on immune response data, protocol was modified again, to require minimum of 3 months and up to 6 months of continuous AHCC supplementation and still required at least 1 month of AHCC beyond first negative result. A total of 10 HR-HPV+ women were enrolled on study and received AHCC 3g daily. All participants had confirmed HR-HPV infections for >2 years and was confirmed again at time of enrollment. Specific HR-HPV strain typing was not financially feasible in the pilot studies. Patient demographic information is summarized in Table 2. Two patient had received AHCC 3g daily on an empty stomach for 5 weeks without a HR-HPV negative response and as per initial protocol design was taken off study which was prior to protocol modifications when minimum AHCC supplementation was extended up to 3 months based on immune response data. Two patients had received AHCC 3g daily on an empty stomach for 2 months without a HR-HPV negative response and were taken off study prior to achieving an IFNβ level < 25 pg/mL. Based on immune response data the duration of AHCC supplementation was extended to up to 6 months. The remaining six patients had received a minimum of 3 months and up to 6 months of AHCC 3g once daily on an empty stomach (= 1 h before meals or 2 h after). Of these six patients, four (66.7%) patients were able to achieve a durable response represented by INF-β level decreasing then staying <25 pg/mL and confirmed no HR-HPV DNA for >30 days off supplementation (*p* < 0.05). IFN-β immune response is summarized in Figure 4A that showed IFN-β levels below level of 25 pg/mL was associated with a successful, durable clearance of persistent, HR-HPV infections. All patients tolerated the AHCC 3g daily on an empty stomach supplementation well with no reported side effects.

**Table 2:** Summary of patient demographics for AHCC pilot studies.

**Demographic**	**AHCC 3g Pilot Study** **(*N* = 10)**	**AHCC 1g pilot study** **(*N* = 10)**
Age	44.6 yr. (±8.8 yr.)	44.9 yr. (±12.6 yr.)
Race	All white	Black (1) White (6) Asian (3)
Ethnicity	Hispanic/Latino (2) Non-Hispanic/Latino (8)	Hispanic/Latino (1) Non-Hispanic/Latino (9)
BMI	26.6 (±4.5)	21.3 (±3.5)
Number of sexual partners	20 (±28)	9 (±7.2)

A separate AHCC 1g follow up pilot study was initiated to determine if a lower dose of AHCC supplementation would also be effective. Patients received AHCC 1g daily on an empty stomach for at least 6 and up to 8 months. Ten women with confirmed persistent HR-HPV infections for >2 years were enrolled. Patient demographics are summarized in Table 2. One patient was taken off the study due to non-compliance with the study protocol, of the remaining nine patients completing the study there were 4 of 9 (44%) patients that had confirmed HR-HPV clearance after 7 months of AHCC 1g supplementation daily on an empty stomach. Again IFNβ suppression <25 pg/mL was confirmed as marker for successful clearance of HR-HPV infection. (Figure 4B) All patients tolerated the AHCC 1g supplementation daily on an empty stomach well with no reported side effects.

## Discussion

The presented bench-to-bedside research provides step-wise data to support the hypothesis that AHCC supplementation modulates host immune system, specifically via suppression of elevated IFNβ levels, to effectively clear chronic, persistent HR-HPV infections. After observing elimination of HR-HPV *in vitro* in the panel of human cervical cancer cell lines, animal studies were completed that also demonstrated successful, durable elimination of HR-HPV after completing AHCC supplementation. Finally, in two “proof of concept” pilot studies of daily AHCC supplementation successful elimination of HR-HPV was achieved that was durable response. Both the animal and human data suggests the mechanism AHCC supplementation supports the host immune system to clear HPV infections is attributed to the modulation of the expression and signaling of IFNβ that is known to be elevated in chronic viral infections (16, 17).

In previous CMV mouse model studies have demonstrated that IFNβ, type I interferon, is associated with virulence of the infection with significantly elevated levels in chronic/persistent viral infections (16, 17). The elevated levels of the IFNβ suppress the release/production of type II interferon (IFNγ) necessary to clear viral infections (16, 17). Hence, the suppression of IFNβ resulted in clearance of the CMV infection (16, 17). HR-HPV, like many viruses such as CMV, has evolved strategies to counteract interferon (IFN) signaling pathways. Specifically, interferon regulatory factors (IRFs) can promote cell immunity as well as promote oncogenesis pathway in response to a variety of extracellular signals (18, 19). Specifically, IRF-2 has been shown to activate HR-HPV E6/E7 gene expression and promote oncogenesis pathway (20). However, eventually in persistent infections, IFNα/β also induce IRF-1 that will help sustain persistent viral gene expression (21, 22). Lace et al. have confirmed that elevated IFNβ levels will induce IRF-1 and IRF-2 promoting HPV16 persistent infections (23). Recently two independent research teams evaluating lymphocytic choriomeningitis virus (LCMV) persistent infections have demonstrated that suppression of chronic IFNβ signaling can reset the host immunity and enable control and clearance of persistent viral infections (16, 17). This research presented here is the first to define that it is actually this mechanism of suppression of IFNβ (type I interferon) by AHCC led to an upregulation of IFNγ (type II interferon) and ultimate clearance of persistent HR-HPV infections.

There are some limitations to data presented. First, in the preclinical setting there is no established HR-HPV animal model which has also been a challenge for in the pre-clinical development of HPV vaccines. As a surrogate a human tumor infected with HR-HPV was employed to determine if the HR-HPV infection could be cleared with AHCC supplementation and was durable response after AHCC supplementation was stopped. In addition, in both the pre-clinical and clinical studies it would be ideal to confirm eradication with quantitative PCR methods as well as evaluate other immune markers such as CD8+ T-cells or natural killer (NK) cells but due to limited funding and being exploratory study, an exhaustive immune panel evaluation could not be completed. However, in the ongoing phase II confirmation study these studies are being evaluated.

There are limited effective treatment options for clearing HR-HPV infections; majority being local treatment modalities is to alleviate symptoms and remove symptomatic lesions that often reoccur. Fortunately, most HR-HPV infections are cleared within 6–18 months on their own without interventions; only ~10% of women will suffer from persistent HR-HPV infections (15, 24). To date, there is no readily available effective systemic interventions to clear HR-HPV infections. Currently prevention of HR-HPV infections with the HPV vaccine nine-valent product before exposure to HPV has demonstrated the best potential for eliminating HR-HPV infections. However, these vaccines have little benefit in those already infected with HR-HPV infections.

This bench to bedsides studies are the first preliminary study to demonstrate AHCC supplementation modulates the host immune response to effectively clear of HR-HPV infections. Similar to other chronic viral infections IFNβ suppression was confirmed as marker of durable clearance of HR-HPV infection. AHCC 3g achieved response slightly faster and more consistently than the AHCC 1g. These preliminary findings are very encouraging and await confirmation from the ongoing phase II randomized, double-blinded, placebo controlled study in 50 women with confirmed HR-HPV+ infections. Futures studies will focus on the potential role for AHCC supplementation in combination with chemotherapy for the treatment of HR-HPV-related cancers.

## Data Availability

The datasets generated for this study are available on request to the corresponding author.

## Author Contributions

JS contributed to conceptualization, data curation, formal analysis, funding acquisition, investigation, methodology, project administration, supervision, writing—original draft and editing. LM and AG contributed to data curation, investigation, formal analysis, methodology, validation, writing—original draft, review and editing. BR and MB contributed to data curation, investigation, writing—review and editing. JF contributed to methodology, investigation, writing—review and editing. JL and TB contributed to investigation, writing—review and editing. YB and RO contributed to methodology, supervision, writing—review and editing.

### Conflict of Interest Statement

JS has been principal investigator on unrestricted research grants from Amino Up Chemical Company to the institution for preliminary preclinical studies. The remaining authors declare that the research was conducted in the absence of any commercial or financial relationships that could be construed as a potential conflict of interest.

## References

[1] American Cancer Society Global Burden of Cancer in Women: Current Status, Trends, and Interventions. Available online at: https://www.cancer.org/content/dam/cancer-org/research/cancer-facts-and-statistics/global-cancer-facts-and-figures/global-burden-of-cancer-in-women.pdf (Accessed July 9, 2018).

[2] Lombard I Vincent-Salomon A Zafrani B de la Rochefordiere A Clough K Favre M. Human papillomavirus genotype as a major determinant of the course of cervical cancer. J Clin Oncol. (1998) 16:2613–9. 10.1200/JCO.1998.16.8.2613 9704710

[3] Harris RWC Brinton LA Cowdell RH Skegg DC Smith PG Vessey MP Characteristics of women with dysplasia or carcinoma *in situ* of the cervix uteri. Br J Cancer. (1980) 42:359–69. 10.1038/bjc.1980.246 7426342 PMC2010409

[4] Furumoto H Irahara M Human papillomavirus (HPV) and cervical cancer. J Med Invest. (2002) 49:124–33. 12323001

[5] Munoz N Bosch FX de Sanjose S Herreor R Castellsague X Shah KV. Epidemiologic classification of human papillomavirus types associated with cervical cancer. N Engl J Med. (2003) 348:518–27. 10.1056/NEJMoa021641 12571259

[6] Andrei G Snoeck R Piette J Delvenne P DeClercq E. Antiproliferative effects of acyclic nucleoside phosphonates on human papillomavirus (HPV)-harboring cell lines compared with HPV-negative cell lines. Oncol Res. (1998) 10:523–31. 10338155

[7] Andrei G Snoeck R Schols D De Clercq E. Induction of apoptosis by cidofovir in human papillomavirus (HPV)-positive cells. Oncol Res. (2000) 12:397–408. 10.3727/096504001108747855 11697818

[8] Schiffman M Wheeler CM Castle PE. Human papillomavirus DNA remains detectable longer than related cervical cytologic abnormalities. J Infect Dis. (2002) 186:1169–72. 10.1086/343816 12355370

[9] Uno K Kosuna K Sun B Fujii H Wakame K Chikumaru S Active Hexose Correlated Compound (AHCC) improves immunological parameters and performance status of patients with solid tumors. Biotherapy. (2000) 14:303–9.

[10] Gao Y Zhang D Sun B Fujii H Kosuna K Yin Z. Active hexose correlated compound enhances tumor surveillance through regulating both innate and adaptive immune responses. Cancer Immunol Immunother. (2006) 55:1258–66. 10.1007/s00262-005-0111-9 16362410 PMC11030784

[11] Hirose A Sato E Fujii H Sun B Nishioka H Aruoma OI. The influence of active hexose correlated compound (AHCC) on cisplatin-evoked chemotherapeutic and side effects in tumor-bearing mice. Toxicol Appl Pharmacol. (2007) 222:152–8. 10.1016/j.taap.2007.03.031 17555784

[12] Hunter RJ Fujii H Wakame K Gaikwad A Wolf JK Smith JA Evaluation of active hexose correlated compound (AHCC) in combination with PEGylated liposomal doxorubicin for treatment of ovarian cancer. Int J Appl Res Nat Prod. (2011) 4:6–11. Available online at: http://www.doaj.org/doaj?func=openurl&issn=19406223&genre=journal

[13] Matsui Y Uhara J Satoi S Kaibori M Yamada H Kitade H. Improved prognosis of postoperative hepatocellular carcinoma patients when treated with functional foods: a prospective cohort study. J Hepatol. (2002) 37:78–86. 10.1016/S0168-8278(02)00091-0 12076865

[14] Kawaguchi Y Improved survival of patients with gastric cancer or colon cancer when treated with active hexose correlated compound (AHCC): effect of AHCC on digestive cancer. Nat Med J. (2009) 1:1–6.

[15] Doorbar J. Host control of human papillomavirus infection and disease. Best Prac Res Clin Obstet Gynaecol. (2018) 47:27–41 10.1016/j.bpobgyn.2017.08.001 28919159

[16] Wilson EB Yamada DH Elsaesser H Herskovitz J Deng J Cheng G Blockage of chronic type I interferon signaling to control persistent LCMV infection. Science. (2013) 340:202–7. 10.1126/science.1235208 23580528 PMC3704950

[17] Teijaro JR Ng C Lee AM Sullivan BM Sheehan KC Welch M Persistent LCMC infection is controlled by blockade of type I interferon signaling. Science. (2013) 340:207–11. 10.1126/science.1235214 23580529 PMC3640797

[18] Fujita T Kimura Y Miyamoto M Barsoumain EI Taniguchi T. Induction of endogenous IFN- alpha and IFN-beta genes by a regulatory transcription factor, IRF-1. Nature. (1989) 337:270–2. 10.1038/337270a0 2911367

[19] Honda K Tanguchi T. Toll-like receptor signaling and IRF transcription factors. IUBMB Life. (2006) 58:290–5. 10.1080/15216540600702206 16754320

[20] Lace MJ Anson JR Haugen TH Turek LP. Interferon regulatory factor (IRF)-2 activates the HPV-16 E6-E7 promoter in keratinocytes. Virology. (2010) 399:270–9. 10.1016/j.virol.2009.12.025 20129639

[21] Kimura T Nakayama K Penninger J Kitagawa M Harada H Matsuyama T. Involvement of the IRF-1 transcription factor in antiviral responses to interferons. Science. (1994) 264:1921–4. 10.1126/science.8009222 8009222

[22] Kano A Haruyama T Akaike T Watanabe Y. IRF-1 is an essential mediator in IFN-gamma-induced cell cycle arrest and apoptosis of primary hepatocytes. Biochem Biophys Res Commun. (1999) 257:672–7. 10.1006/bbrc.1999.0276 10208842

[23] Lace MJ Anson JR Klingelhutz AJ Harada H Taniguchi T Bossler AD. Interferon-beta treatment increases human papillomavirus early gene transcription and viral plasmid genome replication by activating regulatory factor (IRF)-1. Carcinogenesis. (2009) 30:1336–44. 10.1093/carcin/bgp150 19541854 PMC7110192

[24] Ho GYF Bierman R Beardsley L Chang CJ Burk RD. Natural history of cervicovaginal papillomavirus infection in young women. N Engl J Med. 338:423–8. 10.1056/NEJM199802123380703 9459645

